# Is magnesium sulfate useful for tracheal intubation? A systematic review of randomized controlled trials

**DOI:** 10.1590/1806-9282.20241237

**Published:** 2025-03-17

**Authors:** Fabiano Timbó Barbosa, Hermann Silva Brito Lima Buarque de Gusmão, Laura Queiroz Teixeira de Albuquerque, Natália de Oliveira Lima, Célio Fernando de Sousa-Rodrigues, Raul Ribeiro de Andrade

**Affiliations:** 1 Federal University of Alagoas, Faculty of Medicine – Maceió (AL), Brazil.; 2 Federal University of Alagoas, Institute of Health and Biological Sciences, Sector of Human Anatomy – Maceió (AL), Brazil.; 3 Federal University of Alagoas, Institute of Health and Biological Sciences – Maceió (AL), Brazil.

## INTRODUCTION

Tracheal intubation is an essential procedure for general anesthesia. It is frequently used in other clinical settings such as intensive care units, emergency departments, and diagnostic tests^1^. It is not free from complications even in patients without factors predicting a difficult airway^2^.

It is already well known that handling the airways leads to physiological changes due to the release of catecholamines. Some pharmacological agents can mitigate complications, prevent physiological changes, and optimize the clinical conditions of tracheal intubation. Magnesium sulfate appears in this scenario due to its properties of cerebral protection, vasodilation, and ability to treat arrhythmias^3^.

Magnesium sulfate has the potential for muscle relaxation and mitigates the release of catecholamines, so it can improve tracheal intubation conditions, reduce complications, and help control physiological changes; however, there is still no formal recommendation for routine use in clinical practice. The aim of this systematic review is to determine the effectiveness and safety of magnesium sulfate compared to neuromuscular blockers for tracheal intubation.

## METHODS

A tutorial was used to conduct this systematic review and meta-analysis^4^. A protocol was recorded in the PROSPERO database (International Prospective Registry of Systematic Reviews: CRD42023473617). We used the PRISMA guidelines to write this paper^5,6^.

The PICO acronym was defined as follows: population (P)—adults over 18 years; intervention (I)—magnesium sulfate; comparators (C)—neuromuscular blockers; and outcomes (O)—effectiveness and safety. Inclusion criteria were as follows: (1) randomized controlled trials (RCTs) that aimed to verify the effect of magnesium sulfate on tracheal intubation, (2) patients with 18 years or older submitted to general anesthesia, and (3) magnesium sulfate in the intervention group. Exclusion criteria were as follows: (1) incomplete data descriptions, (2) studies that could not be read in full, and (3) duplicated studies.

Six databases were included: Medline via Pubmed, EMBASE, LILACS, SCOPUS, the Cochrane Library, and Google. Databases were searched until February 2024 (search strategies are available at https://shre.ink/magnesiumdata). The reference lists of studies included in the meta-analysis were also searched. There were no language restrictions. Google was used in place of the SIGLE database because it is no longer available to access gray literature.

Two authors assessed titles and abstracts identified by all search strategies. This process was carried out separately. Subsequently, RCTs that appeared to answer our review question were requested to read in full. Disagreements were discussed and resolved by a consensus meeting. The same process was adopted to conduct the risk of bias assessment.

A standardized form was developed, and a pilot test was conducted before collecting data. This process was conducted by one reviewer. A second reviewer confirmed data extracted from the included studies. Data regarding the method, risk of bias, and outcomes were collected. Primary outcomes were as follows: clinical condition of tracheal intubation, time to tracheal intubation, hemodynamic responses, adverse effects, complications, and incidence of adverse effects. Hemodynamic responses of interest were as follows: heart rate, systolic, and diastolic arterial pressure. Complementary data were as follows: participants characteristics (ASA classification), clinical scenario (emergency, surgery, and ICU), Mallampati index, and magnesium sulfate dosage. Authors from RCTs selected were contacted via email to address missing data.

Two reviewers assessed the risk of bias during the data extraction process. We used the Cochrane RoB2 tool^7^. It assesses the randomization process, deviations from intended interventions, missing outcome data, measurement of outcomes, and selection of reported results. Standardized judgments were used to categorize each domain as low risk, some concerns, or high risk (6). Overall was used in this process as an item. We considered some topics in the overall analysis: the description of the funding source, statistical flaws, and sample size. Discrepancies were resolved by consensus meeting.

For continuous outcomes, we used the mean and standard deviation to calculate the weighted mean difference (WMD). For dichotomous outcomes, we used the risk ratio (RR) or risk difference (RD) when no event was described. We considered the random effect model (REM) and 95% confidence interval (CI) for all analyses. Heterogeneity was considered through the Chi-square and I-square tests (substantial heterogeneity>50%) (7). We considered an alpha level for the Chi-square test below 0.1 to demonstrate significant heterogeneity. The RevMan version 5 (the Cochrane collaboration) software was used.

The GRADE approach (Grading of Recommendations Assessment, Development, and Evaluation) was used to assess the certainty of a body of evidence.

## RESULTS

**Figure 1:**
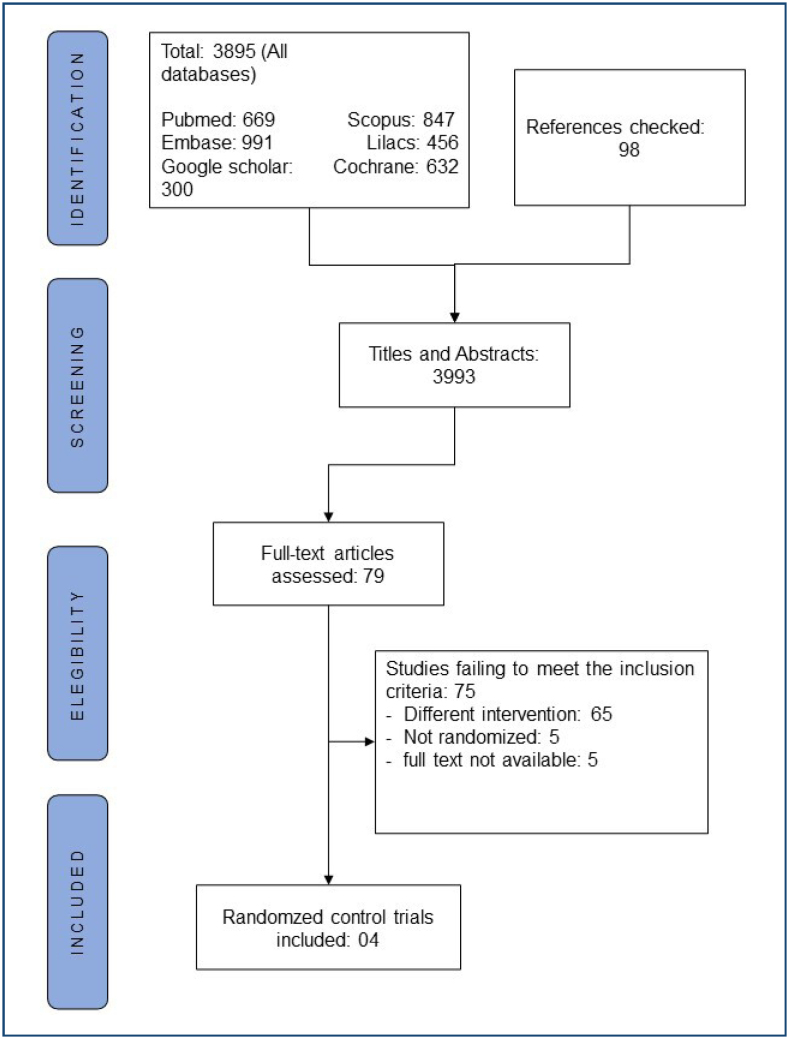
Flowchart of the selection process.

The process for selecting papers is shown in Figure 1. We identified 3,865 records from all databases. We excluded 3,816 after screening the titles and abstracts, and then 79 papers were identified to be read in full. After full-text reading, 75 papers were excluded. The reasons for exclusions were as follows: different interventions (65), inability to get full text (5), and other types of studies (5). Finally, we included four studies in our analysis^8-11^. We analyzed 98 titles from the reference lists of these included studies, but we did not find any new paper.

Included studies were from some countries: Morocco^8^, Brazil^9^, and Iran^10,11^. The mean and standard deviation of age in included studies ranged from 26.4±9.85 to 70.2±7.8 years old. Soltani et al.^11^ used magnesium sulfate in three groups with different dosages. Data from group 50 mg/kg were used in this review. All included studies were conducted in elective surgery scenarios. Magnesium sulfate dosages were as follows: 0.3 mL/kg, 15%^8^, and 50 mg/kg^9,11^. One study did not describe the magnesium sulfate dosage^10^. Two studies did not describe the Mallampati index^9,10^.

### Risk of bias

Three included studies were classified as having a low risk of bias^8,9,11^ and one study as having some concerns^10^. The domain selection of the reported outcomes identified the absence of protocol registration in three included articles^8,10,11^. The overall domain identified missing data in some included articles, such as sample size calculation^11^, funding sources^8-11^, and conflict of interest^11^ (available at https://shre.ink/magnesiumdata).

### Meta-analyses

The clinical condition of tracheal intubation was analyzed in all included studies. There were no statistically significant differences (RD=-0.13; 95%CI −0.64 to 0.37; p=0.61; I^2^ = 96%; p<0.0001; 268 participants). Different measurement scales were described in the included studies.

**Figure 2:**
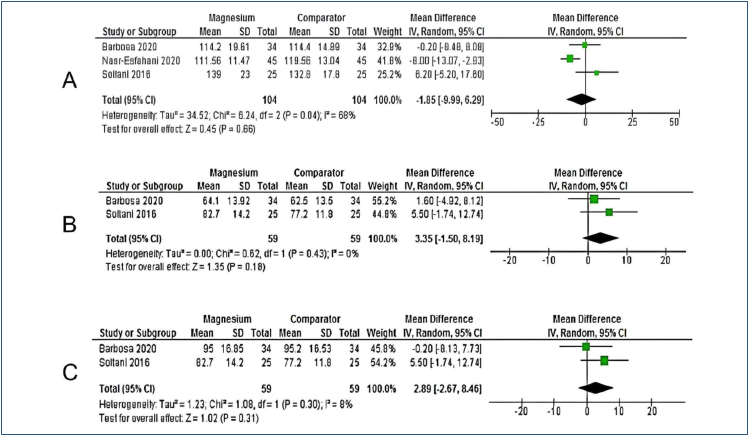
Forest plot. (A) Systolic blood pressure; (B) diastolic blood prussure; (C) heart rate.

The systolic arterial pressure was analyzed in three included studies^9,10,11^. There were no statistically significant differences (WMD=-1.85; 95%CI −9.99 to 6.29; p=0.66; I^2^=68%; p=0.04; 208 participants) (Figure 2A). The diastolic arterial pressure was analyzed in two included studies^9,11^. There were no statistically significant differences (WMD=3.35; 95%CI −1.50 to 8.19; p=0.18; I^2^=0%; p=0.43; 118 participants) (Figure 2B). The heart rate was analyzed in two included studies^9,11^. There were no statistically significant differences (WMD=2.89; 95%CI −2.67 to 8.46; p=0.31; I^2^=8%; p=0.30; 118 participants) (Figure 2C).

The time to tracheal intubation^11^ and incidence of adverse effects^9^ were analyzed only in one study. Adverse effects and complications were not contemplated in the included studies.

### Sensitivity analysis

The clinical condition of tracheal intubation and systolic arterial pressure presented significant heterogeneities. Two studies were responsible for statistical heterogeneity in clinical conditions of tracheal intubation^8,9^. However, there was no statistically significant difference when data from these studies were excluded (RD=-0.12; 95%CI −0.28 to 0.05; p=0.17). The different ways of measuring intubation conditions may have been responsible for the statistical heterogeneity between studies. One study was responsible for statistical heterogeneity in systolic arterial pressure^10^. However, there was no statistically significant difference when data from this study were excluded (WMD=2.01; 95%CI −4.69 to 8.71; p=0.56). We did not identify clinical heterogeneity from this analysis due to the poor reporting of participant details in the studies excluded from the analysis. We contacted the study authors by email but did not receive a response.

### Grading of Recommendations Assessment, Development, and Evaluation system analysis

The force of evidence was considered low in the RCTs about diastolic blood pressure, heart rate, and the clinical conditions of tracheal intubation. However, the systolic blood pressure outcome was very low (available at https://shre.ink/magnesiumdata).

## DISCUSSION

In this systematic review, we synthesized four RCTs including 268 participants under tracheal intubation with magnesium sulfate compared to neuromuscular blockers or saline. The results did not show the superiority of magnesium sulfate compared to neuromuscular blockers for tracheal intubation. We cannot pool the results for time to tracheal intubation, incidence of adverse effects, and complications.

Good clinical conditions of tracheal intubation occurred in 35% (47/134). We believe that the use of magnesium sulfate would bring benefits due to its potential for muscle relaxation^12^; however, our rate showed that less than half of patients had benefit. The idea of using magnesium in this clinical context is new, so it is possible that its actions as a muscle relaxant are not fully understood. It is possible to list some topics that were not described in the included studies, such as the blood magnesium level of the participants before the intravenous infusion, the time elapsed between the infusion and muscle relaxation, and the best combination of intravenous agents for anesthetic induction.

Hemodynamic responses were analyzed in three studies, and there were no statistically significant differences^9,10,11^. The usual dosage in clinical practice is 30 mg/kg over 30 s intravenously; however, researchers usually combine with neuromuscular blockers^12^. In this systematic review, we considered magnesium sulfate with or without these agents, so differences between inclusion criteria can lead to different results.

Future studies should take into account some outcomes to elucidate the effectiveness and safety of magnesium sulfate for tracheal intubation, such as time to tracheal intubation, adverse effects, and complications. It is important to emphasize that the authors of RCTs demonstrate their considerations regarding the sample size calculation and the description of their methodological criteria. The adequate sample size ensuring adequate statistical power associated with a description compatible with a low risk of bias may be sufficient to answer the question of this systematic review.

The findings of this systematic review do not allow us to say that magnesium sulfate provides good conditions for tracheal intubation associated with hemodynamic stability, so the patient's individual conditions and the anesthetist's previous practice should guide their choice in daily clinical practice.

This systematic review has some limitations. First, the heterogeneity was substantial. We explored some sources to identify causes of heterogeneity such as different ways of measuring clinical conditions of tracheal intubations, participant characteristics, data reported only in graphs, and reports from patients with difficult intubations. We contacted the authors to solve our doubts. Second, the small sample size in the included studies may lead to inaccurate results. We used the topic overall in the Cochrane risk of bias tool to assess the impact of sample size. Only one study presented and followed the sample size calculation^9^. In addition, the absence of some outcomes in the included studies limited our analysis. All limitations identified may impact our meta-analysis, and our results should be seen with caution.

## CONCLUSION

The available evidence was low or very low to determine the effectiveness and safety of magnesium sulfate compared to neuromuscular blockers for tracheal intubation. Significant heterogeneity and the absence of some outcomes demonstrate that our results are not definitive.
